# Seipin oligomers can interact directly with AGPAT2 and lipin 1, physically scaffolding critical regulators of adipogenesis

**DOI:** 10.1016/j.molmet.2014.12.013

**Published:** 2015-01-06

**Authors:** Md. Mesbah Uddin Talukder, M.F. Michelle Sim, Stephen O'Rahilly, J. Michael Edwardson, Justin J. Rochford

**Affiliations:** 1 Department of Pharmacology, University of Cambridge, Cambridge CB2 1PD, UK; 2 University of Cambridge Metabolic Research Laboratories, Institute of Metabolic Science, Addenbrooke's Hospital, Cambridge CB2 0QQ, UK; 3 Rowett Institute of Nutrition and Health, Institute of Medical Sciences, University of Aberdeen, Foresterhill, Aberdeen AB25 2ZD, UK

**Keywords:** Lipodystrophy, BSCL2, AGPAT2, Lipin, Seipin, Lipid synthesis, Adipocyte, Adipogenesis, Atomic force microscopy (AFM)

## Abstract

**Objective:**

Disruption of the genes encoding either seipin or 1-acylglycerol-3-phosphate O-acyltransferase 2 (AGPAT2) causes severe congenital generalized lipodystrophy (CGL) in humans. However, the function of seipin in adipogenesis remains poorly defined. We demonstrated recently that seipin can bind the key adipogenic phosphatidic acid (PA) phosphatase lipin 1 and that seipin forms stable dodecamers. As AGPAT2 generates PA, the substrate for lipin 1, we investigated whether seipin might bind both enzymes of this lipid biosynthetic pathway, which is required for adipogenesis to occur.

**Methods:**

We employed co-immunoprecipitation and immunofluorescence methods to determine whether seipin can interact with AGPAT2 and the consequences of this in developing adipocytes. Atomic force microscopy was used to determine whether these interactions involved direct association of the proteins and to define the molecular architecture of these complexes.

**Results:**

Our data reveal that seipin can bind AGPAT2 during adipogenesis and that stabilizing this interaction during adipogenesis can increase the nuclear accumulation of PPARγ. Both AGPAT2 and lipin 1 can directly associate with seipin dodecamers, and a single seipin complex can simultaneously bind both AGPAT2 and lipin with a defined orientation.

**Conclusions:**

Our study provides the first direct molecular link between seipin and AGPAT2, two proteins whose disruption causes CGL. Moreover, it provides the first example of an interaction between seipin and another protein that causally influences a key aspect of adipogenesis. Together our data suggest that the critical role of seipin in adipogenesis may involve its capacity to juxtapose important regulators of this process in a multi-protein complex.

## 1 Introduction

Homozygous disruption of either *BSCL2* or *AGPAT2*, encoding seipin and 1-acylglycerol-3-phosphate O-acyltransferase 2 (AGPAT2) respectively, causes a very severe congenital generalized lipodystrophy (CGL). Affected individuals display a striking lack of adipose tissue and suffer severe metabolic disease [1–4]. Both seipin and AGPAT2 have critical cell-autonomous roles in adipogenesis, and the loss of these functions is likely to explain the failure of patients to develop adipose tissue [5–9].

AGPAT2 converts lysophosphatidic acid (LPA) to phosphatidic acid (PA), a key step in the synthesis of both phospholipids and triacylglycerol (TG). Loss of AGPAT2 in preadipocytes causes a failure to induce adipogenic gene expression before TG accumulation would normally occur during adipocyte differentiation [6,9]. Indeed, there is now strong evidence that the loss of pro-adipogenic signalling lipids or the accumulation of inhibitory lipid signals, and not impaired TG synthesis *per se*, is the primary cause of failed adipogenesis in AGPAT2 deficiency [9].

Despite its critical role in human adipose tissue development, the molecular role of seipin in adipogenesis remains uncertain. Several studies have suggested an evolutionarily conserved role for seipin in lipid droplet biogenesis [10–18]. Seipin-deficient mouse embryonic fibroblasts (MEFs) display increased lipolysis and consequent loss of lipid accumulation [19,20]; however, the molecular basis of this observation remains uncertain. Seipin has also been shown to bind the adaptor protein 14-3-3β in developing adipocytes and may influence adipogenesis via cytoskeletal remodelling [21]. However, the specific loss of 14-3-3β during adipogenesis causes reduced lipid accumulation, but not the failure of adipogenic gene expression observed with seipin disruption. A recent study has also demonstrated that seipin can bind the sarco/endoplasmic reticulum Ca^2+^-ATPase, SERCA, and may modulate its activity [22]. However, it is not clear how or if this may influence adipogenesis.

We demonstrated previously that seipin can bind the key adipogenic PA phosphatase lipin 1 [23]. The loss of seipin in developing adipocytes reduces the quantity of lipin bound to the endoplasmic reticulum (ER), where seipin resides, and increases the levels of PA [23]. The effects of lipin or seipin loss on the induction of genes regulating adipogenesis are remarkably similar, and so the binding of lipin at the ER may represent a critical role for seipin in adipocyte development.

We also showed recently, that human seipin forms homo-oligomers of twelve subunits [24], similar to the nine-subunit homo-oligomers reported for its orthologue in yeast [25]. This led us to speculate that seipin dodecamers might provide a docking nexus around which several molecules could be arranged. AGPAT2 lies immediately upstream of lipin 1 in the same lipid biosynthetic pathway, and loss of either enzyme or seipin inhibits adipogenesis in a similar manner. Hence, we investigated whether seipin might act to juxtapose AGPAT2 and lipin 1.

## 2 Materials and methods

### 2.1 Cell culture

HEK293 cells were cultured in DMEM containing 10% FBS and transiently transfected using Fugene 6 transfection reagent (Promega) as in [24]. 3T3-L1 preadipocytes were cultured and differentiated as previously described [23]. Differentiating adipocytes were transiently transfected as in [23] using Lipofectamine LTX (Invitrogen). For BiFC experiments, HEK293 or 3T3-L1 cells were grown on glass coverslips, transfected with S-Yn, Yn-S, A-Yc and Yc-A plasmids using Fugene 6 (Roche) or Lipofectamine LTX, respectively. HEK293 cells were incubated at 37 °C for 4 h, at 32 °C for 20 h then at 30 °C for 2 h. Cells were then fixed or harvested, permeabilized, blocked and probed as previously described [23]. For AFM experiments, tsA 201 cells (a subclone of HEK293) were grown in DMEM containing 10% FBS and transiently transfected using calcium phosphate precipitation. A total of 250 μg of DNA was used to transfect cells in 5 × 162 cm^2^ culture flasks, and cells were harvested 48 h later in a total volume of 9 ml. Approximately 8 ml of a Triton X-100 extract of the cells was incubated with 50 μl of appropriate immunoresin in order to immunoprecipitate proteins for AFM analysis.

### 2.2 Constructs

Constructs to express FLAG-seipin, FLAG-AGPAT2 and FLAG-seipin-Myc were generated in the pCMV3xFLAG vector (Sigma–Aldrich). Seipin-Myc and AGPAT2-Myc were in the pcDNA3.1/Myc-His vector (LifeTechnologies), and HA-lipin 1 was cloned in pCMV (Sigma–Aldrich) as previously described [7,8,23,24]. Fusion constructs for BiFC experiments were generated essentially as in [23]. Briefly, the N-terminal (1–158) fragment of YFP was inserted downstream or upstream of seipin in pCMV3xFLAG to generate S-Yn and Yn-S fusion constructs, respectively. The C-terminal (155–239) fragment was amplified and inserted downstream or upstream of Myc-tagged AGPAT2 in pcDNA3.1/Myc-His to make A-Yc and Yc-A fusion constructs, respectively.

### 2.3 Immunoprecipitations and immunoblotting

Lysates and anti-FLAG or anti-Myc immunoprecipitates were prepared as described in [23,24]. Briefly, 48 h after transfection HEK293 cells were lysed in n-octyl-β-d-glucopyranoside (ODG) lysis buffer comprising 50 mM ODG, 50 mM Tris, pH 6.8, 150 mM NaCl, 1 mM EDTA supplemented with protease inhibitors (Complete EDTA-free, Roche Applied Science) and phosphatase inhibitor cocktails (Sigma–Aldrich). All subsequent steps were performed on ice. Cells were sonicated at medium intensity for two cycles of 30 s, incubated on ice for 20 min, centrifuged at 16,000 g for 10 min at 4 °C and supernatants retained. Lysate containing 1 mg of protein was added to 30 μl of anti-FLAG-agarose beads (Sigma–Aldrich) pre-equilibrated with ODG lysis buffer and ODG lysis buffer added to a final volume of 800 μl. Tubes were rotated gently for 2 h at 4 °C. Following centrifugation (8,200 g for 30 s at 4 °C) supernatants were removed and beads washed three times with ODG lysis buffer. Excess lysis buffer was removed after the final wash. FLAG-tagged proteins were eluted from beads by addition of 100 μl of 200 ng/μl 3xFLAG peptide (Sigma–Aldrich) in TBS (50 mM Tris–HCl, pH 7.4, 150 mM NaCl) and incubated with gentle rotation for 30 min at 4 °C. Beads were then centrifuged and supernatant transferred to a new tube.

Lithium dodecyl sulfate (LDS) sample buffer (Invitrogen) was added to 20 μg of lysate and 20 μl of IP samples. Lysate and IP samples to be probed for seipin were not heated prior to loading to prevent aggregation. All other samples were heated to 95 °C for 5 min. Samples were separated by SDS-PAGE and transferred onto nitrocellulose membranes. Immunoblots were probed with antibodies to Myc (clone 4A6 Millipore), FLAG (Sigma–Aldrich), HA (Covance HA.11 clone 16B12), lipin 1 (generously provided by Symeon Siniossoglou, CIMR, Cambridge, UK) or calnexin (Abcam), followed by horseradish peroxidase (HRP)-linked secondary antibodies (Cell Signaling & Thermo Scientific). Proteins were visualized by ECL (GE Healthcare) and imaged using the Bio-Rad ChemiDoc system.

### 2.4 BiFC and immunofluorescence

Antibodies used were as described for immunoblotting. Highly cross-adsorbed Alexa Fluor anti-mouse 594, anti-rabbit 594 or anti-mouse 488 secondary antibodies were used for detection (Invitrogen). Images were acquired and BiFC quantification was as described in [23].

### 2.5 AFM imaging of isolated proteins

Transfected tsA 201 cells were lysed and proteins isolated and eluted from anti-Myc, anti-HA or anti-FLAG agarose as described in [26]. Isolated proteins were imaged using ‘tapping’ mode in air, as described previously [24]. Particle heights and diameters were measured manually by the Nanoscope software and used to calculate the molecular volume of each particle using the equation

(1) $$V_{m}=\left(\pi h/6\right)\left(3r^{2}+h^{2}\right)$$

where *h* is the particle height and *r* is the radius [27]. This equation assumes that the adsorbed particles adopt the form of a spherical cap. Molecular volume based on molecular mass was calculated using the equation

(2) $$V_{c}=\left(M_{0}/N_{0}\right)\left(V_{1}+dV_{2}\right)$$

where *M*_0_ is the molecular mass, *N*_0_ is Avogadro's number, *V*_1_ and *V*_2_ are the partial specific volumes of particle (0.74 cm^3^/g) and water (1 cm^3^/g), respectively, and *d* is the extent of protein hydration (taken as 0.4 g water/g protein).

### 2.6 Statistical analysis

Quantitative data are represented as mean ± SEM. For statistical analysis the differences between groups were examined with ANOVA followed by a Tukey's post-hoc test. P < 0.05 was considered statistically significant.

## 3 Results

### 3.1 Seipin can associate with AGPAT2

**Figure 1:**
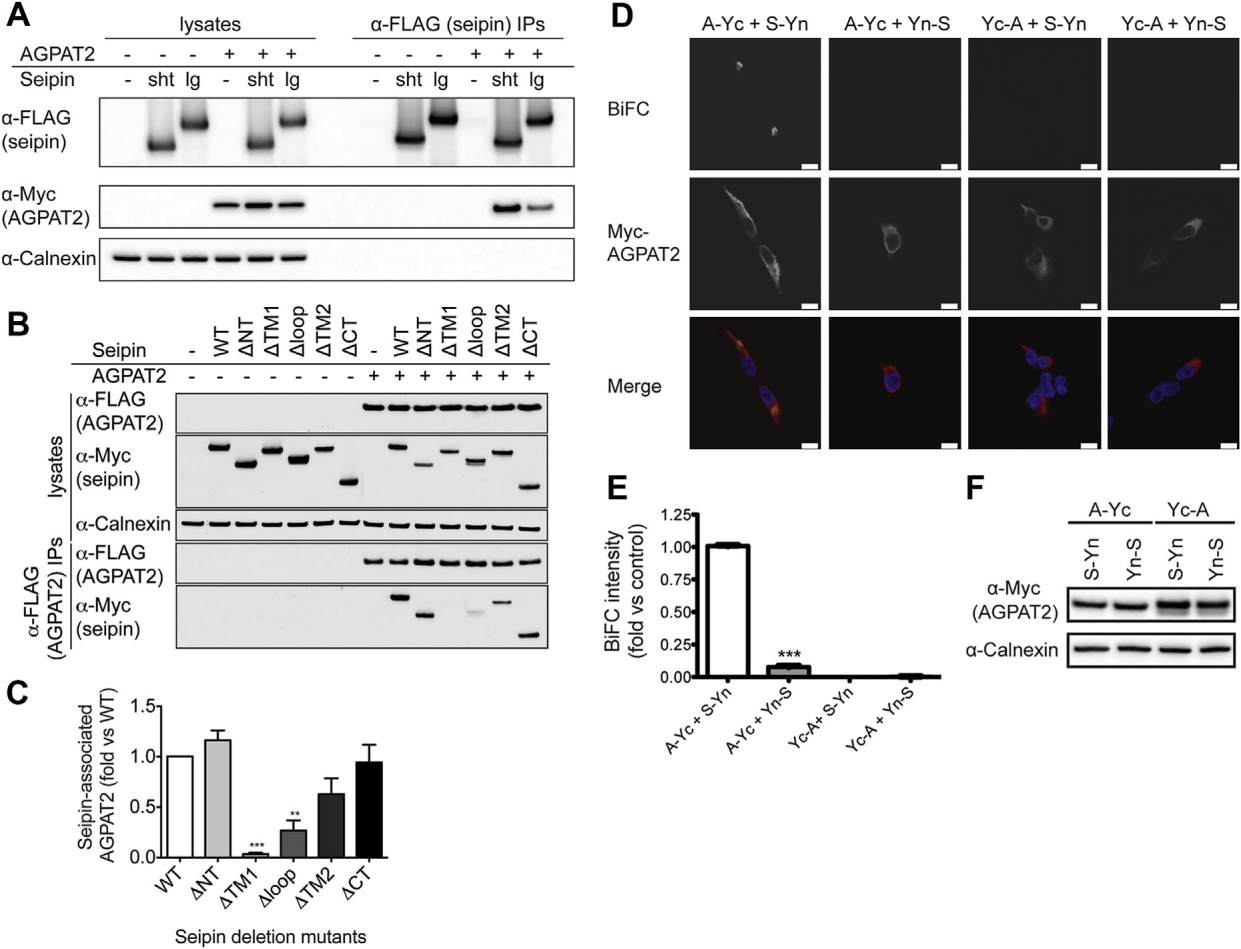
Seipin can associate with AGPAT2 in intact cells. (A) HEK293 cells were transfected with AGPAT2-Myc in the presence or absence of either the short (sht) or long (lg) translation of FLAG-seipin. Lysates and anti-FLAG immunoprecipitated proteins were separated by SDS-PAGE and immunoblotted with antibodies to FLAG and Myc. Lysates were probed for calnexin as a loading control. (B) HEK293 cells were transfected with FLAG-AGPAT2 in the absence or presence of either wild-type Myc-seipin (WT) or mutants lacking the N terminus (ΔNT), first transmembrane domain (ΔTM1), ER luminal loop region (ΔLP), second transmembrane domain (ΔTM2), or the C terminus (ΔCT). Lysates or anti-FLAG immunoprecipitates were immunoblotted for FLAG, Myc and calnexin. (C) Quantification of the interaction of mutant vs wild-type seipin from replicate experiments shown in (B). Data are means ± SEM (n = 3). ** indicates p < 0.01, *** indicates p < 0.001 *versus* co-immunoprecipitation with wild-type seipin. (D) HEK293 cells were co-transfected with constructs in which the N-terminal fragment of YFP was fused to the N terminus (Yn-S) or the C terminus (S-Yn) of seipin and constructs in which the C-terminal fragment of YFP was fused to either the N terminus (Yc-A) or the C terminus (A-Yc) of AGPAT2-Myc. Following a temperature shift to induce the formation of reconstituted YFP, cells were fixed and stained for AGPAT2-Myc (red) and DAPI to label nuclei. The direct interaction between seipin and AGPAT2 is indicated by the presence of a YFP signal (yellow). Scale bars, 10 μm. (E) Quantified BiFC signal intensity. Errors are SEM (n = 3). *** indicates difference from A-Yc/S-Yn (p < 0.001). (F) Representative immunoblot using anti-Myc antibody to detect Yc-AGPAT2 and AGPAT2-Yc expression, showing calnexin as a loading control.

In co-immunoprecipitation experiments, Myc-tagged human AGPAT2 could be detected in anti-FLAG immunoprecipitates of HEK293 cells where AGPAT-Myc was co-expressed with FLAG-tagged seipin (Figure 1A). The interaction was observed with both the short 398-amino acid translation of seipin and the long form of the protein containing an additional 64 amino acids at the N terminus. To define the regions of seipin important for this interaction, we used mutant forms of seipin lacking either the cytosolic N terminus (ΔNT), first transmembrane domain (ΔTM1), ER luminal loop (Δloop), second transmembrane domain (ΔTM2) or cytosolic C terminus (ΔCT). Deletion of the ER luminal loop of seipin significantly impaired its interaction with AGPAT2, whilst almost no AGPAT2 could be immunoprecipitated with the ΔTM1 form of seipin (Figure 1B,C). Although the ΔTM1 mutant of seipin may have altered topology, previous studies have shown that this mutant is mostly membrane associated with N and C termini exposed to the cytoplasm like the wild-type protein [28]. Overall, this result indicates that the evolutionarily conserved luminal loop of seipin and the first transmembrane region may be important for its interaction with AGPAT2.

To investigate the interaction between seipin and AGPAT2 in intact cells, we employed bimolecular fluorescence complementation (BiFC) analysis. The C-terminal portion of YFP (Yc) was fused to either the N or C terminus of AGPAT2 to generate Yc-AGPAT2 or AGPAT2-Yc, respectively. Similarly, the N-terminal portion of YFP (Yn) was fused to the N or C terminus of seipin to generate Yn-seipin and seipin-Yn, respectively. Consistent with an association between AGPAT2 and seipin, we observed YFP fluorescence when AGPAT2-Yc and seipin-Yn were co-expressed (Figure 1D,E). However, no YFP fluorescence was observed with other combinations of seipin and AGPAT2 fusions, despite equivalent expression of AGPAT2 (Figure 1F). This result argues strongly against a non-specific aggregation of these proteins, which would not be sensitive to the position of the half-YFP proteins in the fusions.

### 3.2 Seipin and AGPAT2 can interact during early adipogenesis and potentiate this process

**Figure 2:**
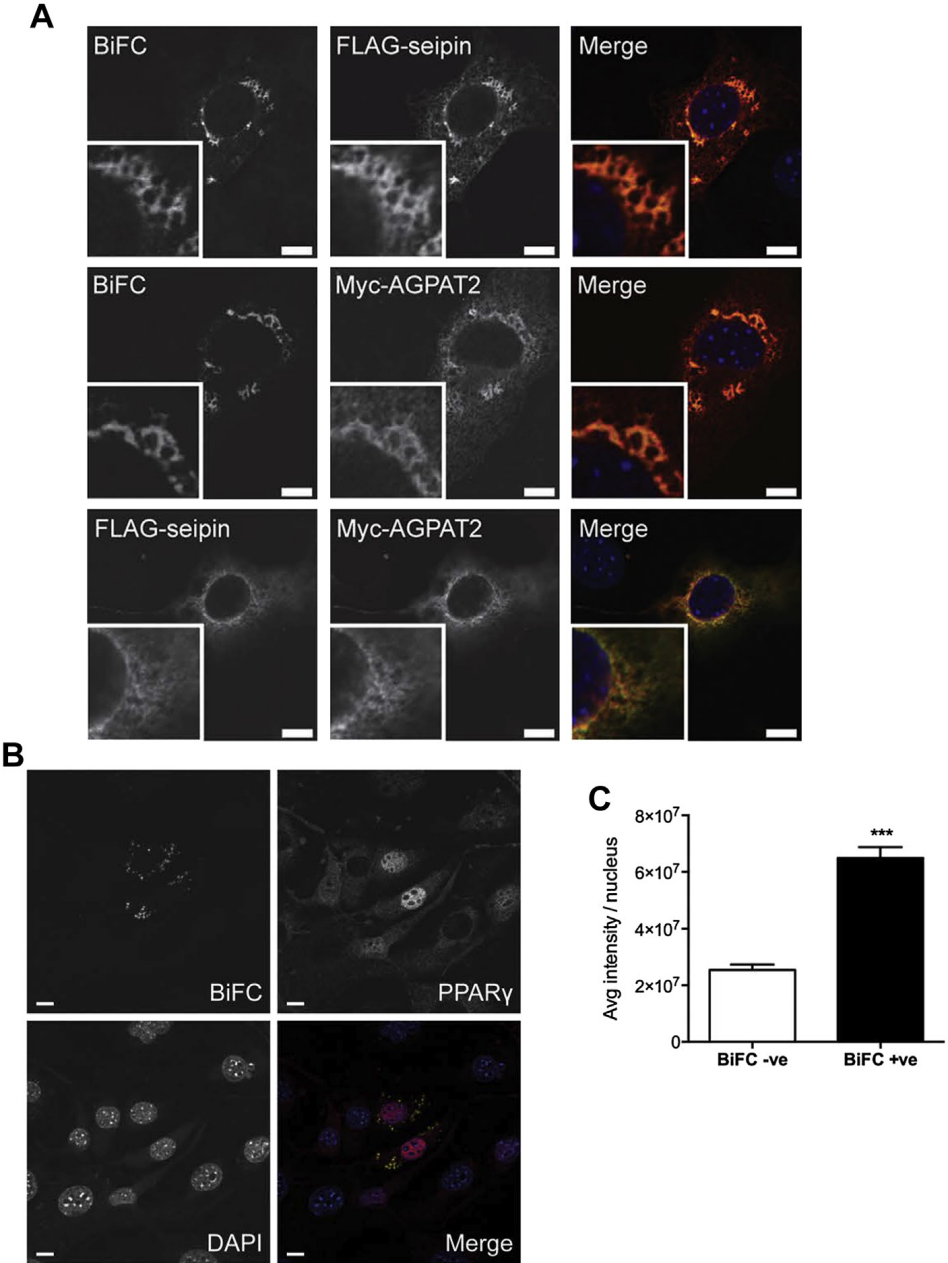

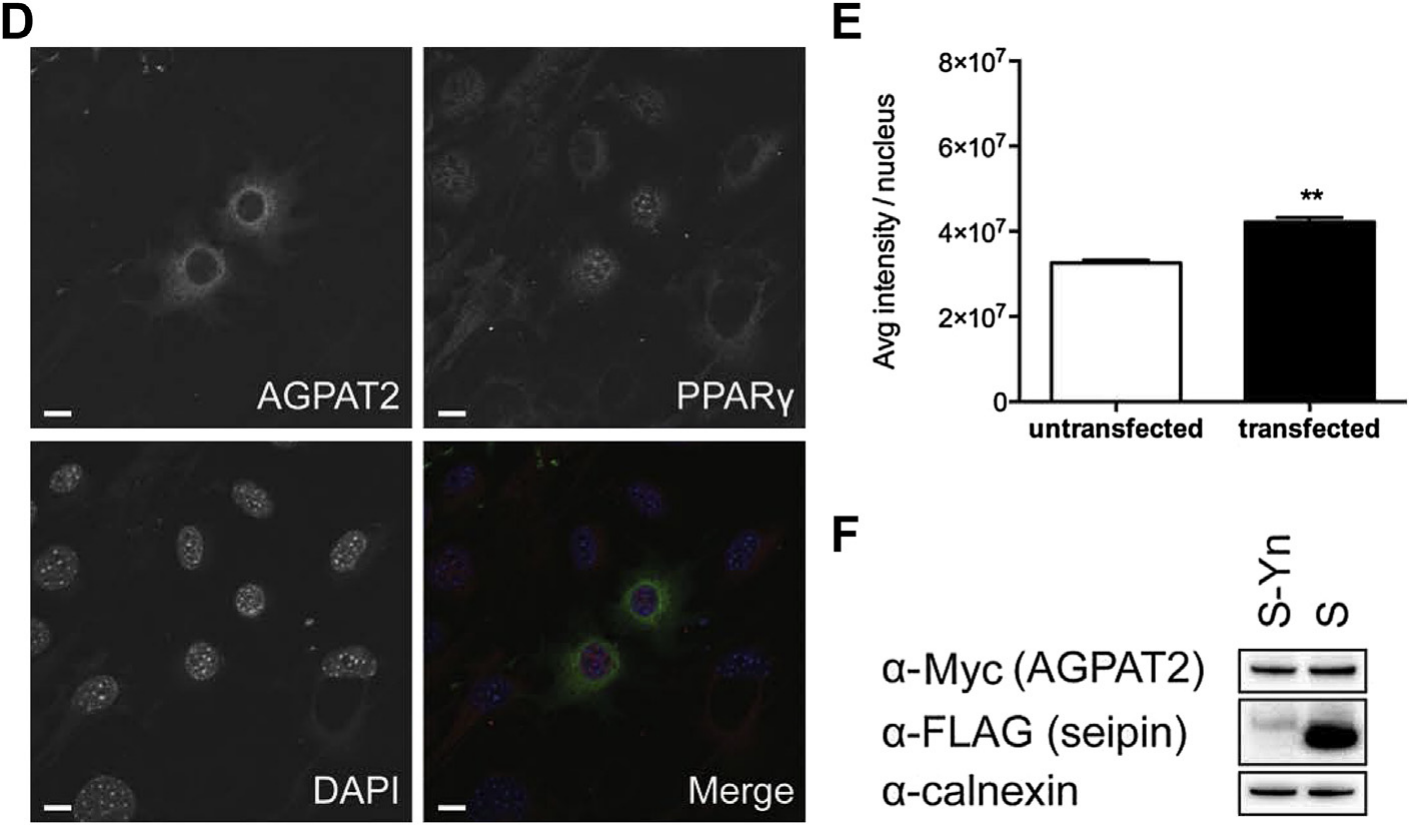
Seipin can interact with AGPAT2 in differentiating adipocytes and potentiate adipogenesis. (A) 3T3-L1 preadipocytes were induced to differentiate and immediately co-transfected with FLAG-seipin-Yn and Myc-AGPAT2-Yc. Cells were fixed after three days of differentiation, and anti-FLAG or anti-Myc antibodies in combination with Alexa Fluor 594 secondary antibodies were used to immunodetect FLAG-seipin-Yn or Myc-AGPAT2-Yc, respectively, as indicated. Identically transfected cells were used to co-immunostain for FLAG-seipin-Yn and Myc-AGPAT2-Yc (lower panels). Note that these cells were not temperature shifted, preventing the formation of YFP which would otherwise confound the Alexa Fluor488 fluorescence of the secondary antibody used to detect the FLAG epitope. Scale bars, 10 μm. (B) 3T3-L1 preadipocytes were induced to differentiate and immediately co-transfected with FLAG-seipin-Yn and Myc-AGPAT2-Yc, as in (A). Following a temperature shift to induce the stable formation of reconstituted YFP, cells were fixed at day 3 of differentiation and stained for PPARγ (red) and DAPI to label nuclei. The direct interaction between seipin and AGPAT2 is indicated by the presence of a YFP signal (yellow). Scale bars, 10 μm. (C) Quantified PPARγ signal intensity in BiFC-negative (BiFC −ve) and BiFC-positive (BiFC +ve) cell nuclei. Intensity of PPARγ staining was determined in 100 nuclei each of BiFC-negative and BiFC-positive cells in 3 separate experiments, and mean intensity values were determined for each experiment. Errors are SEM (n = 3). *** indicates difference from BiFC-positive cell nuclei (p < 0.001). (D) 3T3-L1 preadipocytes were induced to differentiate, immediately co-transfected with FLAG-seipin (with no Yn) and Myc-AGPAT2-Yc and otherwise treated exactly as those in (B). Cells were fixed at day 3 of differentiation and stained for Myc to identify transfected cells, PPARγ and DAPI to label nuclei. Scale bars, 10 μm. (E) Quantified PPARγ signal intensity in the nuclei of untransfected *versus* transfected cells. Intensity of PPARγ staining was determined in 100 nuclei each of transfected *versus* untransfected cells in 3 separate experiments and mean intensity values were determined for each experiment. Errors are SEM (n = 3). ** indicates difference from untransfected cell nuclei (p < 0.01). (F) Lysates of cells transfected with Myc-AGPAT2-Yc and either FLAG-seipin-Yn or FLAG-seipin as in (B) and (D) were immunoblotted for AGPAT2 and seipin using anti-Myc and anti-FLAG antibodies, respectively, to determine the relative levels of each protein. Samples were also probed for calnexin as a loading control.

To investigate the interaction between seipin and AGPAT2 in developing adipocytes, seipin-Yn and AGPAT2-Yc were co-transfected into post-confluent 3T3-L1 adipocytes and these cells were induced to differentiate. A BiFC signal could be clearly detected at the ER of these cells at day 3 of differentiation, and this signal co-localized significantly with either AGPAT2 or seipin expressed in these cells (Figure 2A). In similarly transfected cells not subjected to the temperature shift, in order to prevent the formation of confounding YFP fluorescence, we observed significant co-localization of the seipin-Yn and AGPAT2-Yc proteins (Figure 2A, lower panels). No reconstitution of BiFC signal occurred if seipin-Yn was expressed with Yc-AGPAT2 in identically treated differentiating 3T3-L1 adipocytes, indicating that the association of seipin and AGPAT2 in these cells occurs with a specific orientation (Figure S1A).

The formation of the reconstituted YFP protein is irreversible and, as a consequence, the seipin and AGPAT2 to which the half-YFP proteins are fused will become stably associated. We next determined whether this stable complex of AGPAT2 and seipin could influence adipogenesis. Differentiating 3T3-L1 preadipocytes were transfected with AGPAT2-Yc and seipin-Yn constructs, and the nuclear expression of the key pro-adipogenic transcription factor PPARγ was used as a marker of adipogenesis. In preadipocytes expressing AGPAT2-Yc and seipin-Yn that had been differentiated for 3 days, the intensity of staining for nuclear PPARγ was significantly higher in cells positive for BiFC signal than in BiFC-negative cells in the same cultures (Figure 2B,C). This did not result merely from the overexpression of these proteins, as identically treated cells co-expressing AGPAT2-Yc and FLAG-seipin (not bearing Yn) displayed only very modestly increased nuclear PPARγ staining compared with untransfected cells in the same cultures (Figure 2D,E and S1B). This was despite equivalent expression of AGPAT2-Yc in both cases, and more robust expression of FLAG-seipin than FLAG-seipin-Yn (Figure 2F). Overall, this result strongly implies that stabilizing the complex of AGPAT2 and seipin can functionally alter the rate of differentiation in intact preadipocytes during early adipogenesis.

### 3.3 AFM imaging reveals that AGPAT2 directly interacts with seipin dodecamers

**Figure 3:**
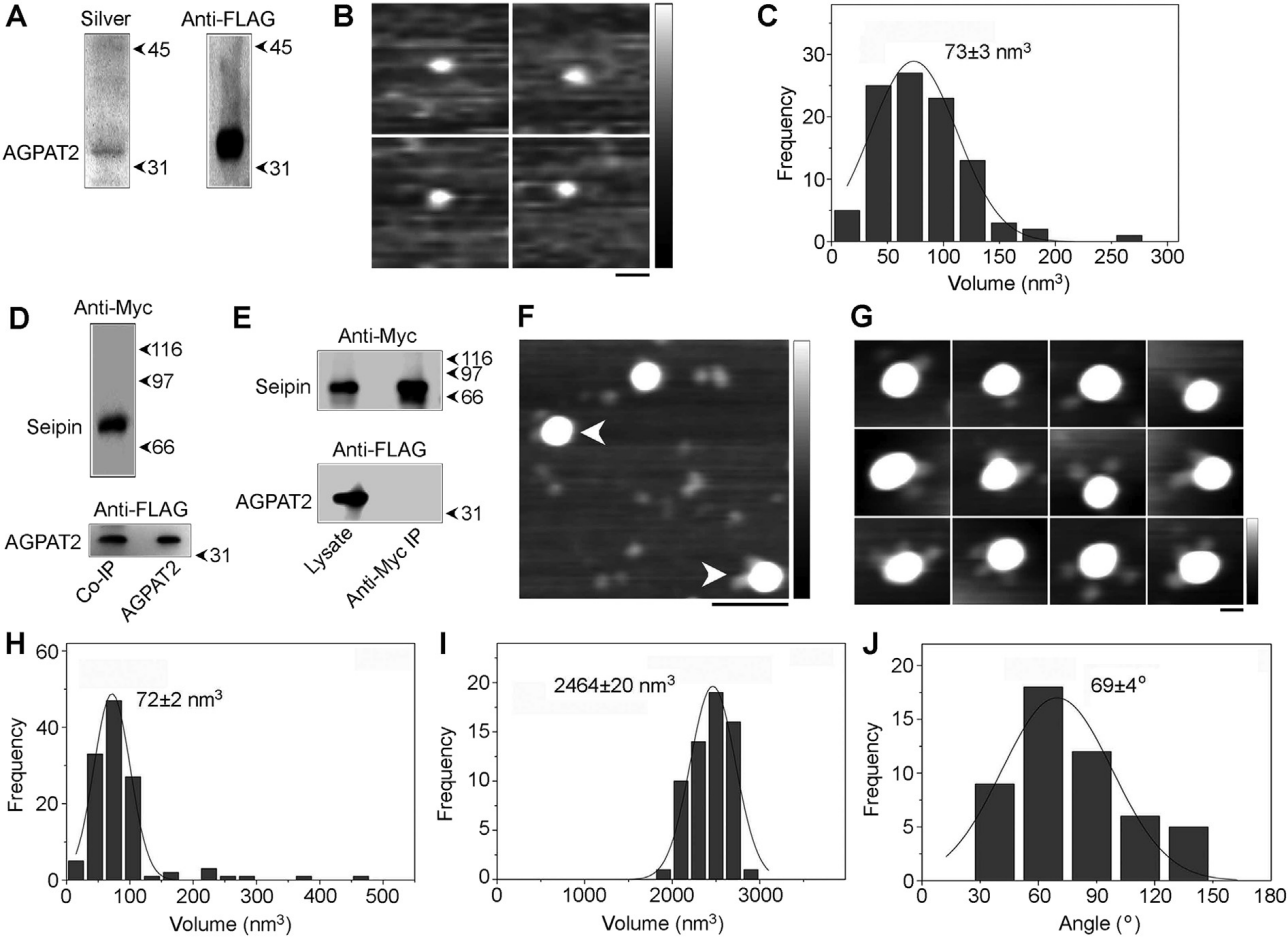
AFM analysis of AGPAT2 and its interaction with seipin. (A) FLAG-AGPAT2 was expressed in tsA 201 cells and isolated using anti-FLAG immunoaffinity chromatography. Isolated protein was analyzed by SDS-PAGE followed by either silver staining (left panel) or immunoblotting using an anti-FLAG antibody (right panel). The position of AGPAT2 is indicated at the left, and molecular mass markers (kDa) are shown at the right. (B) Gallery of zoomed AFM images showing individual isolated AGPAT2 particles. Scale bar, 25 nm; height scale, 0–1 nm. (C) Frequency distribution of volumes of the AGPAT2 particles. The curve indicates the fitted Gaussian function. The peak of the distribution (±SEM) is indicated. (D) FLAG-seipin-Myc and FLAG-AGPAT2 were co-expressed in tsA 201 cells and proteins were isolated using anti-Myc immunoaffinity chromatography. Isolated protein was analyzed by SDS-PAGE followed by immunoblotting using either anti-Myc (top panel) or anti-FLAG (bottom panel) antibodies. The positions of seipin and AGPAT2 are indicated at the left, and molecular mass markers (kDa) are shown at the right. The bottom panel shows immunoprecipitated AGPAT2 and AGPAT2 expressed alone. (E) Control experiment in which two batches of cells separately expressing either FLAG-seipin-Myc or FLAG-AGPAT2 were mixed immediately before solubilization, followed by anti-Myc immunoaffinity chromatography. Samples of both total cell lysate and immunoprecipitate were immunoblotted using either anti-Myc (top panel) or anti-FLAG (bottom panel) antibodies. (F) Low-magnification AFM image of isolated proteins. Arrowheads indicate large particles (seipin), each decorated by two smaller particles (AGPAT2). Scale bar, 100 nm; height scale, 0–2 nm. (G) Gallery of zoomed images showing seipin particles decorated by either one (top panels), two (center panels) or three (bottom panels) AGPAT2 particles. Scale bar, 25 nm; height scale, 0–2 nm. (H) Frequency distribution of volumes of the smaller (AGPAT2) particles. The curve indicates the fitted Gaussian function. The peak of the distribution (±SEM) is indicated. (I) Frequency distribution of volumes of the larger (seipin) particles. (J) Frequency distribution of angles between pairs of bound AGPAT2 particles.

We next turned to AFM to investigate the molecular architecture of seipin/AGPAT2 complexes. Following expression in HEK-derived tsA 201 cells, FLAG-AGPAT2 was immunoisolated (Figure 3A), eluted and subjected to AFM imaging (Figure 3B). Analysis of the volumes of AGPAT2 particles revealed a single peak at 73 ± 3 nm^3^ (n = 100) (Figure 3C), close to the expected volume of 64 nm^3^ calculated from the molecular mass (see supplementary methods). When FLAG-AGPAT2 and FLAG-seipin-Myc were co-expressed, both proteins were detected in anti-Myc immunoprecipitates (Figure 3D). No AGPAT2 was present in anti-Myc immunoprecipitates when separately transfected cells were mixed prior to lysis, demonstrating that the association occurs within intact cells (Figure 3E). AFM analysis of the co-immunoprecipitated proteins following elution revealed large particles with smaller, peripherally associated particles (Figure 3F). Zoomed images of representative complexes are shown in Figure 3G. The peak molecular volume of the peripheral particles (72 ± 2 nm^3^; n = 122; Figure 3H) was almost identical to that of AGPAT2 alone (Figure 3C). The peak volume of the core of the complex was 2464 ± 20 nm^3^ (n = 61; Figure 3I), almost identical to the volume of 2394 nm^3^ that we have previously reported for seipin dodecamers [24]. Interestingly, we observed a number of multiple decorations of the seipin dodecamers by AGPAT2 (Figure 3G). Specifically, 12.8% (50/391) of seipin particles were doubly decorated by AGPAT2, and 1.0% (4/391) were triply decorated. In contrast, no multiple decorations were seen when seipin was expressed alone (157 particles). The frequency distribution of angles between pairs of bound AGPAT2 molecules had a peak at 69° ± 4° (n = 50; Figure 3J). This result implies a preference for the binding of any two AGPAT2 molecules to seipin subunits separated by a single seipin subunit, given that the toroidal, dodecameric arrangement of seipin oligomers should generate an angle of 30° separation between individual subunits [24]. Together, these analyses show that AGPAT2 can associate directly with dodecamers of seipin, whilst the precise molecular architecture of this interaction indicates a specific and highly ordered interaction.

### 3.4 Seipin dodecamers can directly bind to lipin 1

**Figure 4:**
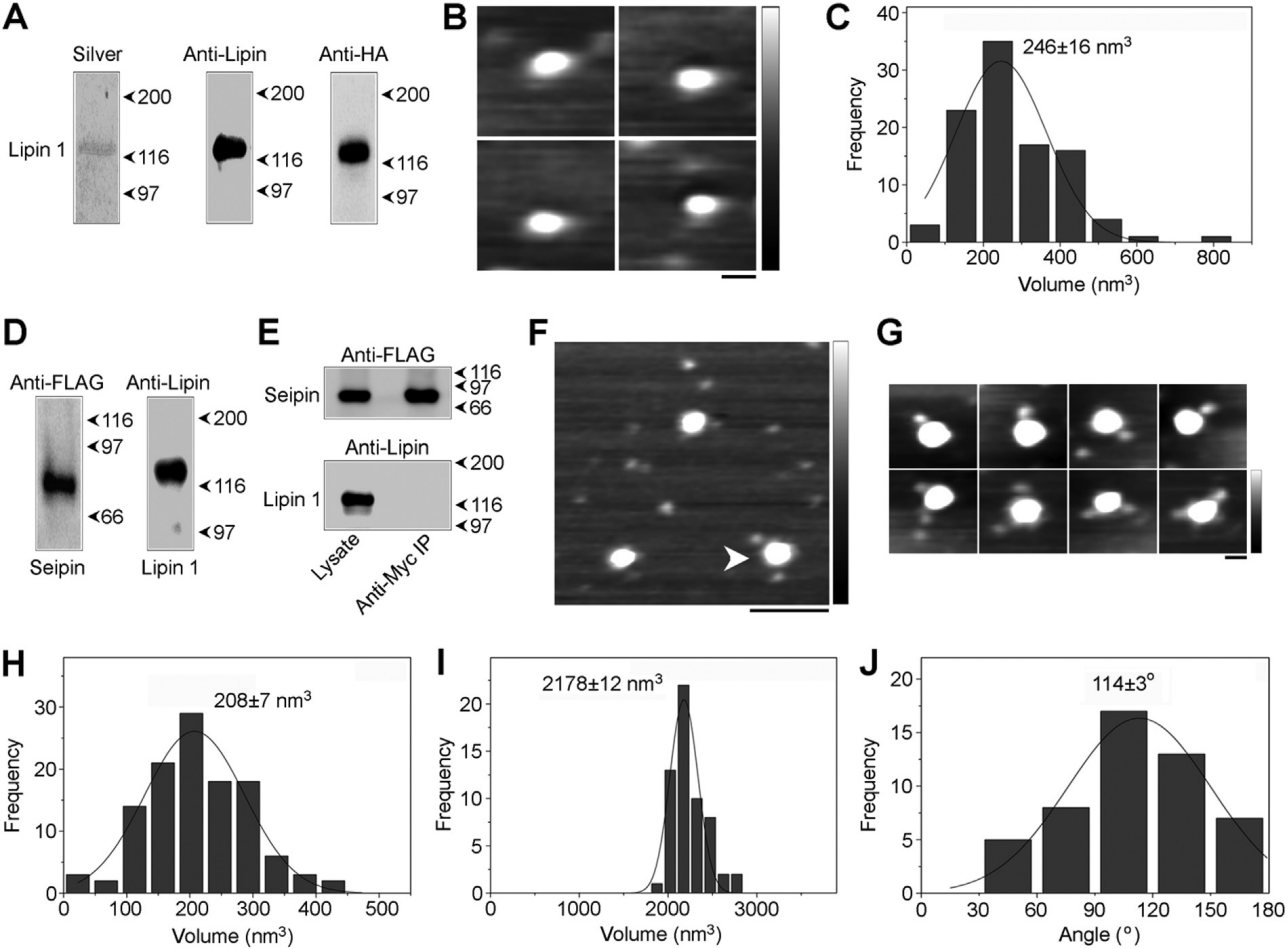
AFM analysis of lipin 1 and its interaction with seipin. (A) HA-lipin 1α was expressed in tsA 201 cells and isolated using anti-HA immunoaffinity chromatography. Isolated protein was analyzed by SDS-PAGE followed by either silver staining (left panel), or immunoblotting using either an anti-lipin 1 antibody (center panel) or an anti-HA antibody (right panel). The position of lipin 1 is indicated at the left, and molecular mass markers (kDa) are shown at the right. (B) Gallery of zoomed AFM images showing individual isolated lipin 1α particles. Scale bar, 25 nm; height scale, 0–1 nm. (C) Frequency distribution of volumes of the lipin 1α particles. The curve indicates the fitted Gaussian function. The peak of the distribution (±SEM) is indicated. (D) FLAG-seipin-Myc and HA-lipin 1α were co-expressed in tsA 201 cells and proteins were isolated using anti-Myc immunoaffinity chromatography. Isolated protein was analyzed by SDS-PAGE followed by immunoblotting using either anti-FLAG (left panel) or anti-lipin 1 (right panel) antibodies. Molecular mass markers (kDa) are shown at the right. (E) Control experiment in which two batches of cells separately expressing either FLAG-seipin-Myc or HA-lipin 1α were mixed immediately before solubilization, followed by anti-Myc immunoaffinity chromatography. Samples of both total cell lysate and immunoprecipitate were immunoblotted using either anti-FLAG (top panel) or anti-lipin 1 (bottom panel) antibodies. (F) Low-magnification AFM image of isolated proteins. The arrowhead indicates a large particle (seipin) decorated by two smaller particles (lipin 1α). Scale bar, 100 nm; height scale, 0–2 nm. (G) Gallery of zoomed images showing seipin particles decorated by either one (top panels) or two (bottom panels) lipin 1 particles. Scale bar, 25 nm; height scale, 0–2 nm. (H) Frequency distribution of volumes of the smaller (lipin 1) particles. The curve indicates the fitted Gaussian function. The peak of the distribution (±SEM) is indicated. (I) Frequency distribution of volumes of the larger (seipin) particles. (J) Frequency distribution of angles between pairs of bound lipin 1 particles.

We next examined the association of lipin 1 with seipin that we have reported previously. HA-tagged lipin 1α isolated from transfected tsA 201 cells (Figure 4A) had a peak volume of 246 ± 16 nm^3^ (n = 100; Figure 4B,C), close to the expected volume of 227 nm^3^, and to the volume previously reported by others on the basis of AFM analysis [29]. FLAG-seipin-Myc and HA-lipin 1α could be co-isolated by anti-Myc immunoaffinity chromatography from co-transfected cells (Figure 4D), but no association was seen when individually transfected cells were mixed prior to lysis (Figure 4E). AFM imaging of the isolated proteins revealed large particles decorated by smaller peripheral particles (Figure 4F). Whilst single decoration events were the most common, double decorations were also observed. We found that 9.0% (50/557) of seipin particles were doubly decorated by lipin 1. Zoomed images of representative complexes are shown in Figure 4G. The peak molecular volume of the peripheral particles was 208 ± 7 nm^3^ (n = 116; Figure 4H), whilst the core particles had a peak volume of 2178 ± 12 nm^3^ (n = 58; Figure 4I). These sizes indicate that the complexes consist of seipin dodecamers with associated lipin 1 monomers. The distribution of angles between pairs of bound lipin 1 molecules had a peak at 114°±3° (n = 50; Figure 4J), suggesting that lipin 1 may preferentially bind to seipin subunits separated by two subunits in the dodecamers, although other configurations are clearly possible. Importantly, this analysis reveals that lipin 1 can bind directly to seipin dodecamers, and confirms that this interaction is specific and organized.

### 3.5 Individual seipin dodecamers can complex both AGPAT2 and lipin 1

**Figure 5:**
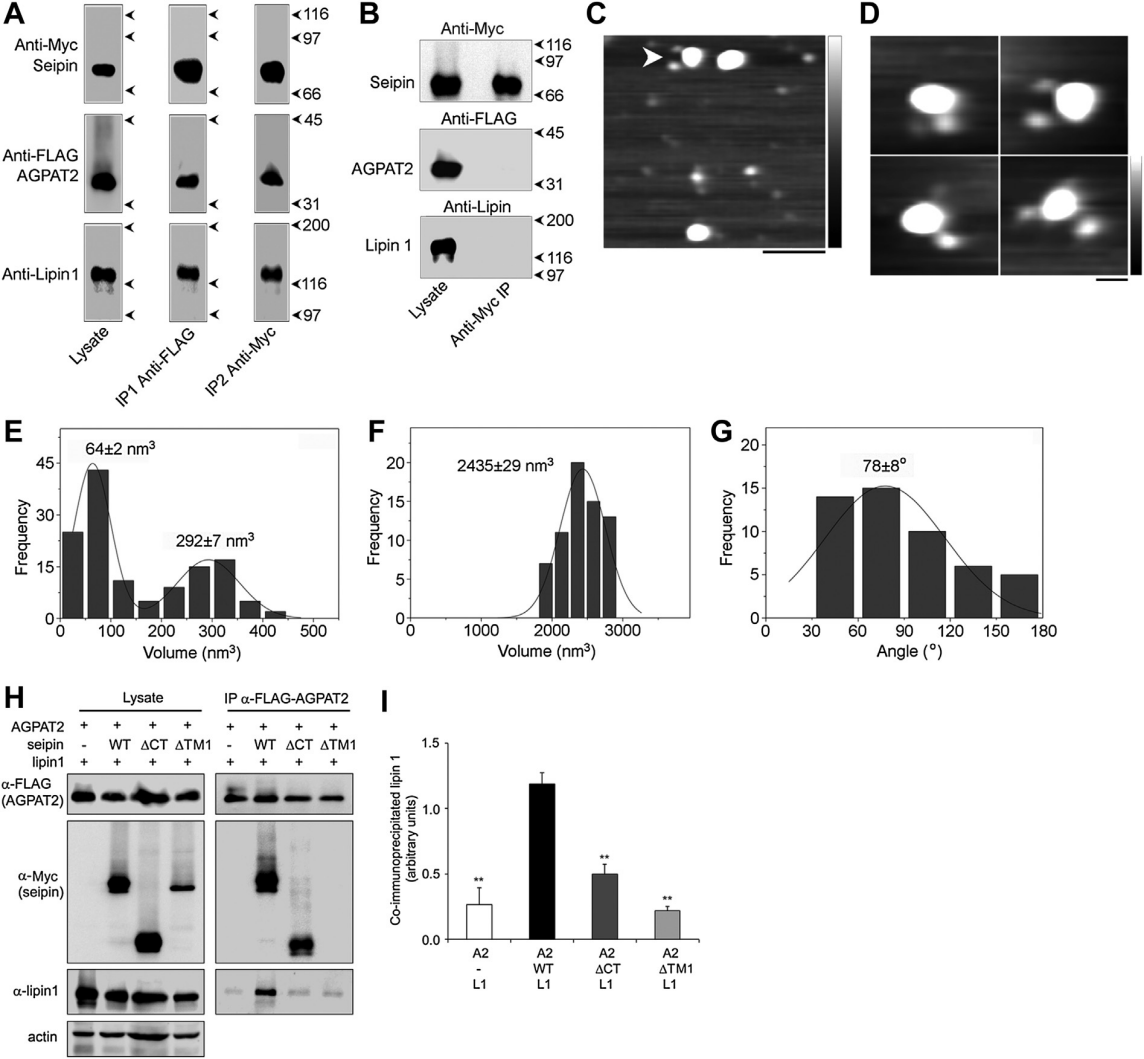
Seipin forms a triple complex with AGPAT2 and lipin 1. (A) Seipin-Myc, FLAG-AGPAT2 and HA-lipin 1α were co-expressed in tsA 201 cells. A detergent extract of the cells was subjected to sequential anti-FLAG (IP1) and anti-Myc (IP2) immunoaffinity chromatography steps. Isolated protein was analyzed by SDS-PAGE followed by immunoblotting using either anti-Myc (seipin; top panels), anti-FLAG (AGPAT2; centre panels) or anti-lipin 1 (bottom panels) antibodies. The positions of the three proteins are indicated at the left, and molecular mass markers (kDa) are shown at the right. (B) Control experiment in which three batches of cells separately expressing either seipin-Myc, FLAG-AGPAT2 or HA-lipin 1α were mixed immediately before solubilization, followed by anti-Myc immunoaffinity chromatography. Samples of both total cell lysate and immunoprecipitate were immunoblotted using either anti-Myc (top panel), anti-FLAG (centre panel) or anti-lipin 1 (bottom panel) antibodies. The positions of seipin, AGPAT2 and lipin 1 are indicated at the left, and molecular mass markers (kDa) are shown at the right. (C) Low-magnification AFM image of proteins isolated using sequential immunoaffinity chromatography, as in (A). The arrowhead indicates a large particle (seipin) decorated by two differently-sized smaller particles (AGPAT2 and lipin 1). Scale bar, 100 nm; height scale, 0–2 nm. (D) Gallery of zoomed images showing seipin particles decorated by two differently-sized smaller particles. Scale bar, 25 nm; height scale, 0–2 nm. (E) Frequency distribution of volumes of the smaller particles (AGPAT2 and lipin 1). The curve indicates the fitted Gaussian functions. The peaks of the distribution (±SEM) are indicated. (F) Frequency distribution of the larger (seipin) particles. (G) Frequency distribution of angles between pairs of bound smaller particles. (H) tsA 201 cells were co-transfected with FLAG-AGPAT2 and HA-lipin 1α in the absence or presence of wild-type seipin (WT) or seipin lacking either the cytoplasmic C-terminus (ΔCT) or the first transmembrane domain (ΔTM1). FLAG-AGPAT2 was immunoprecipitated and lysates or immunoprecipitates (IP) were immunoblotted to determine the levels of FLAG-AGPAT2 and associated Myc-seipin or lipin 1α using anti-FLAG, anti-Myc and anti-lipin 1 antibodies, as indicated. (I) The levels of lipin 1α associated with immunoprecipitated AGPAT2 in the absence or presence of wild-type or mutant seipin proteins were determined in three independent experiments. Data shown are means ± SEM of co-precipitated lipin 1α normalized to levels of co-precipitated AGPAT2. ** indicates difference from levels of lipin 1 associated with AGPAT2 in the presence of WT seipin (p < 0.01).

The significantly different molecular volumes of AGPAT2 and lipin 1 allowed us to use AFM to assess whether seipin dodecamers might simultaneously bind both AGPAT2 and lipin 1. Both seipin-Myc and lipin 1 could be detected in immunoprecipitates of FLAG-AGPAT2 from cells co-transfected with seipin-Myc, FLAG-AGPAT2 and HA-lipin 1α (Figure 5A, IP1). After elution of bound proteins a second, anti-Myc immunoprecipitation was performed to enrich for seipin-Myc-associated AGPAT2. Both FLAG-AGPAT2 and HA-lipin 1α were detected in this second eluate (Figure 5A, IP2), indicating a three-way interaction between seipin, AGPAT2 and lipin 1. Importantly, an anti-Myc IP did not precipitate either FLAG-AGPAT2 or HA-lipin 1α when singly transfected cells were mixed prior to lysis (Figure 5B). AFM analysis of the sequentially purified complexes revealed multiple seipin dodecamers doubly decorated by a large and a small peripheral particle, consistent with the binding of both AGPAT2 and lipin 1 (Figure 5C). Zoomed images are shown in Figure 5D. We found that 10.1% (50/496) of seipin particles were doubly decorated by AGPAT2 plus lipin 1. Volume analysis of the peripheral particles revealed two peaks, at 64 ± 2 nm^3^, consistent with the volume of AGPAT2, and 292 ± 7 nm^3^, consistent with the volume of lipin 1 (n = 132; Figure 5E). The core of the complex had a molecular volume of 2435 ± 29 nm^3^ (n = 66), consistent with that of seipin dodecamers (Figure 5F). Together these data provide strong evidence that both lipin 1 and AGPAT2 can associate with the same seipin oligomer. The distribution of angles between the seipin-associated lipin and AGPAT2 particles had a peak at 78 ± 8° (n = 50; Figure 5G), suggesting that there may be some preference for a selective distribution of these proteins around the seipin oligomer. Interestingly, this arrangement would imply that AGPAT2 and lipin 1 could not be simultaneously bound by the A212P and L91P pathogenic mutants of seipin which we have previously shown do not appropriately form dodecamers and instead typically assemble into tetramers [24]

To determine whether the presence of seipin can facilitate the interaction between AGPAT2 and lipin 1, we co-transfected cells with FLAG-AGPAT2 and lipin 1α in the presence or absence of wild-type and mutant forms of seipin. Almost no lipin 1 could be detected in anti-FLAG immunoprecipitates from cells transfected with only FLAG-AGPAT2 and lipin 1α (Figure 5H). However, co-transfection of wild-type seipin significantly increased the quantity of lipin 1α that could be co-immunoprecipitated with AGPAT2. In contrast, co-transfection of the ΔTM1 or ΔCT forms of seipin, which are unable to interact with AGPAT2 (see Figure 1B,C) or lipin 1 [23], respectively, did not increase the association between AGPAT2 and lipin 1. Quantified data are shown in Figure 5I.

## 4 Discussion

This is the first study to report an association between AGPAT2 and seipin, two proteins whose disruption accounts for the majority of cases of CGL [2,3]. As such, it provides the first direct link between the two forms of severe lipodystrophy caused by mutation of the genes encoding these proteins, and strongly suggests that a common molecular mechanism may underlie several aspects of the development of CGL in these cases.

Using AFM imaging, we have shown that the interaction between AGPAT2 and seipin is direct, as is the association of seipin with lipin 1 that we have described previously. This is important because, alternatively, lipin 1 could have been held at the ER by association with its substrate PA [30,31] or by the CTDNEP1/NEP1-R1 complex, which is orthologous to the yeast Nem1p/Spo7p complex [32–34]. These two mechanisms can increase the membrane association of lipin in other cell types and, in theory, either of these interactions might have been indirectly influenced by seipin. However, our data compellingly demonstrate that a direct interaction can occur making this the most likely means by which seipin influences the quantity of lipin 1 associated with the ER in developing adipocytes. Interestingly, Creutz et al. have recently examined the molecular architecture of lipin 1 using AFM [29]. Consistent with our data, they reported that isolated lipin 1 exists as monomers under the conditions used here. However, when applied to a lipid bilayer, they observed that lipin can subsequently self-assemble into larger oligomers over time. It will be interesting to determine whether the addition of seipin alters the self-assembly of lipin complexes in bilayers and whether multiple seipin/lipin 1 complexes might associate under these conditions. Indeed, although our data demonstrate that seipin can form discrete complexes with lipin and AGPAT2, it remains possible that seipin could also act in concert with other scaffold proteins in larger complexes or raft structures in intact cells.

Our data strongly support a model in which seipin acts as a molecular scaffold that can associate with both AGPAT2 and lipin simultaneously. As we previously observed with seipin and lipin 1, only one orientation of seipin and AGPAT2 BiFC fusion proteins yielded reconstituted YFP fluorescence. In addition, deletion of the luminal loop domain of seipin dramatically reduced its interaction with AGPAT2, despite the fact that this mutant form of seipin is exclusively localized in the ER and appropriately orientated in the ER membrane as is the wild-type protein [23,28]. However, the selective geometry of the complexes observed by AFM provides even greater and very persuasive evidence that the interaction of either AGPAT2 or lipin with seipin represent specific and highly organized associations. This is particularly important in the case of AGPAT2 and seipin, as both proteins are inserted in the ER membrane, theoretically raising the potential for non-specific co-immunoprecipitation.

The triple interaction of both AGPAT2 and lipin 1 with an individual dodecamer of seipin is particularly interesting, and may facilitate more efficient supply of PA to associated lipin 1. In our previous study, we demonstrated that the loss of seipin increased PA levels in developing adipocytes at an early stage of adipogenesis, when AGPAT2, lipin and seipin are all critically required for this process [23]. This is consistent with our model as, in the absence of seipin, PA produced by AGPAT2 would not be efficiently supplied to lipin 1 and so would accumulate. Also consistent with this model, increased PA levels have been reported in other studies of seipin disruption, most recently in the fat of adipose-specific seipin null mice [12,15,35]. We focused in this study on lipin 1, which is critical for adipogenesis in murine cells, but we demonstrated previously that seipin can bind both lipin 1 and lipin 3 [23]. It now appears likely that both of these lipin isoforms may influence adipogenesis [36], perhaps explaining why loss of lipin 1 alone does not cause overt lipodystrophy in humans [37]. Hence, it may be relevant that our previous work implies that seipin would be similarly capable of complexing either lipin 1 or lipin 3 with AGPAT2. Despite their critical importance, lipin 1 and AGPAT2 are weakly expressed during the early stages of adipogenesis and become abundant only as adipocytes mature [6,38,39]. Hence, seipin may be required to increase the localized concentration of AGPAT2 and lipin 1 in domains of the ER membrane given the relatively low abundance of these enzymes. This could facilitate their sequential actions on LPA and PA to generate adipogenic lipid signals during this initial period of adipocyte differentiation. We are currently undertaking detailed lipidomic analyses to define the consequences of seipin loss on lipids in developing adipocytes in order to investigate this possibility further.

Whilst our data show that seipin, AGPAT2 and lipin can interact in a single complex and do so specifically, we acknowledge that it is a feature of AFM that the proteins being examined must be overexpressed. The lack of suitable antibodies to detect murine seipin and AGPAT2 in immunoblots has also limited our ability to examine the interaction of endogenous proteins. As seipin, lipin 1 and AGPAT2 are poorly expressed during early adipogenesis, it may be technically challenging to examine their interactions using the techniques employed here even using reliable antibodies. Nevertheless it is clearly a priority to apply novel techniques, such as tagging the endogenous proteins by knock-in, in combination with fluorescent or enzymatic reporter assays to confirm that the interactions reported here also occur between endogenous proteins in developing adipocytes. Similarly it will be challenging but important to examine whether these interactions occur and are important for adipogenesis *in vivo*. Although difficult, the recent advances in identifying preadipocyte precursor cell populations and genetic models to specifically target these will aid future attempts to do this [40–43].

Our data provide the first evidence that seipin may act as a docking site for multiple proteins. Given its dodecameric arrangement, it could also juxtapose alternative proteins in other processes occurring at the ER membrane. The majority of the metabolic features of congenital seipin disruption, such as hepatic steatosis and insulin resistance, can be primarily attributed to the loss of adipose tissue. However, this does not exclude the potential for important seipin-dependent functions in other tissues, such as the brain where seipin is highly expressed with a defined neuroanatomical distribution [44]. Indeed, seipin loss has been reported to cause anxiety related phenotypes and defective spermatogenesis [45,46], whilst other phenotypes could be masked by the profound metabolic consequences of congenital seipin deficiency. These could result from the loss of seipin's interaction with proteins reported here and elsewhere or by additional, as yet undiscovered, binding partners. Seipin deficiency also causes a paradoxical increase in TG accumulation in a variety of non-adipogenic cells in multiple species [11–18] as well as in mature adipocytes *in vivo* [35]. This phenomenon is difficult to reconcile with the profound ability of seipin loss to inhibit adipogenesis, and thereby TG accumulation, in developing adipocytes, and with our observations that seipin may positively influence lipin activity [5,7,23]. The ability of seipin loss to increase TG accumulation may result from interactions with other proteins that control TG synthesis or hydrolysis. Alternatively, AGPAT2 and lipin 1 may selectively regulate the production of pro-adipogenic signalling lipids, but not TG, whilst bound to seipin at the ER. Lipogenic enzymes have been shown to translocate from the ER to the lipid droplet to promote TG synthesis [47]. Therefore, the loss of seipin could liberate AGPAT2 and lipin 1 from the ER to take part in TG synthesis at the lipid droplet. As such this could both inhibit the production of pro-adipogenic signals in differentiating adipocytes and yet increase the potential for TG synthesis in mature adipocytes and other cells. Indeed transgenic overexpression of seipin in mature adipocytes *in vivo* leads to reduced TG accumulation and decreased adipocyte size, which would also be consistent with this effect [48]. Clearly further studies are required to address these possibilities.

A novel interaction between seipin and the ER calcium pump SERCA has been reported recently, adding to the growing number of seipin binding proteins that have been identified [22]. This is particularly interesting, as BSCL2 patients frequently suffer from cardiomyopathy not seen with AGPAT2 deficiency in BSCL1 patients. It may be that altered SERCA function in cardiomyocytes, influenced by seipin but not AGPAT2, may at least partly underlie this. Other differences also exist between the phenotype of BSCL1 and BSCL2 patients, notably that the adipose tissue loss in BSCL2 is typically more severe. This may be due to the loss of additional signals influenced by seipin but not AGPAT2, partial redundancy amongst AGPAT isoforms or the differences in tissue distribution of the two proteins. Further studies that delineate the consequences of seipin or AGPAT2 loss during adipogenesis are likely to shed light on which of these may occur.

It has been shown that both AGPAT2 and lipin 1 are required for the activation of PPARγ during adipogenesis [9,49]. Similarly, loss of seipin during adipogenesis leads to an inability to sustain PPARγ expression [5,7,18,19], whilst PPARγ agonists can partially ameliorate the phenotype of lipodystrophic seipin null mice [20]. These studies are all consistent with our hypothesis that the capacity of seipin to co-regulate AGPAT2 and lipin might contribute to its critical role in adipocyte formation. Our data demonstrate that increasing the interaction between seipin and AGPAT2 can increase the nuclear accumulation of PPARγ. This is only a single indicator of adipogenesis and so the first step in delineating how seipin may control adipogenesis. Nonetheless, our study is the first to show that selectively modulating the interaction of seipin with a binding partner can directly influence a key parameter of adipogenesis. Evidently, further more detailed work is required to determine the importance of the seipin/AGPAT/lipin complex in adipocyte differentiation and to further define the molecular pathways by which these proteins regulate adipogenesis.

## 5 Conclusions

Overall, our data provide the first demonstration that seipin dodecamers can act as scaffolds for multiple proteins and can simultaneously bind both AGPAT2 and lipin 1, two critical regulators of adipogenesis. Moreover our work provides the first direct, physical and mechanistic link between seipin and AGPAT2, the two proteins whose disruption most commonly causes severe generalized lipodystrophy in humans.

## Appendix A Supplementary data

Figure S1. (A) As a control for the BiFC analysis shown in Figure 2A 3T3-L1 preadipocytes were induced to differentiate and immediately co-transfected with FLAG-seipin-Yn and Yc-myc-AGPAT2. Cells were fixed after three days of differentiation, and anti-FLAG or anti-Myc antibodies in combination with Alexa Fluor 594 secondary antibodies were used to immunodetect seipin-Yn or AGPAT2-Yc respectively. No reconstituted YFP signal could be detected in any cells. In the lower panels cells were co-immunostained for FLAG-seipin-Yn and Yc-myc-AGPAT2-Yc. Scale bars, 10 μm. (B) control for cells transfected for Figure 2D,E. 3T3-L1 preadipocytes were induced to differentiate and immediately co-transfected with FLAG-seipin and myc-AGPAT2-Yc as in Figure 2D. Cells were fixed at day 3 of differentiation and stained for FLAG-seipin, myc-AGPAT2-Yc and DAPI to label nuclei. All cells expressing myc-AGPAT2-Yc co-expressed FLAG-seipin. Scale bars, 10 μm.
