# Supplementary figures and images for: Seipin oligomers can interact directly with AGPAT2 and lipin 1, physically scaffolding critical regulators of adipogenesis
Source: Mol Metab. 2015 Jan 6;4(3):199–209. doi: 10.1016/j.molmet.2014.12.013 (PMC4338318; doi:10.1016/j.molmet.2014.12.013)

# Talukder Figure S1

**A**

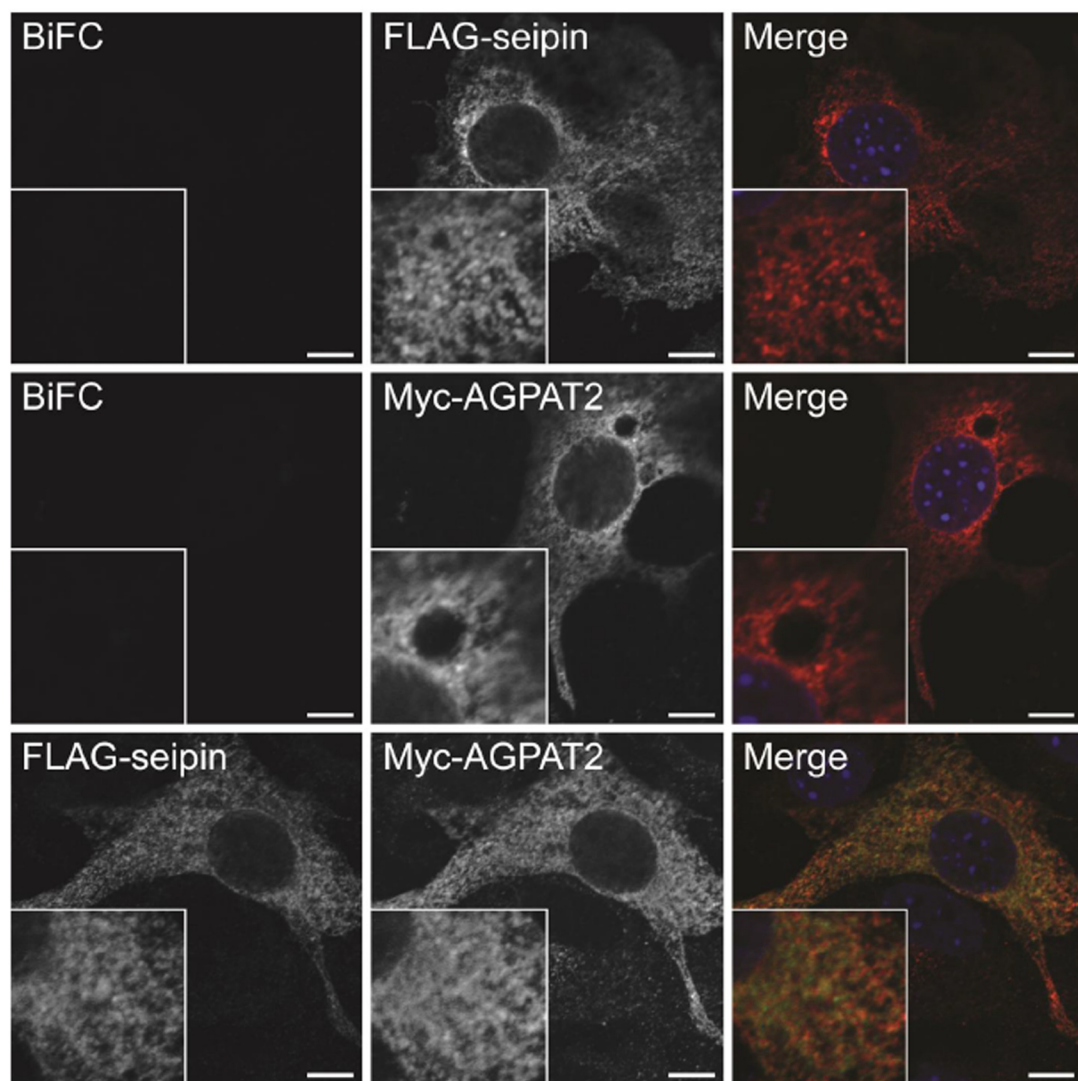

**B**

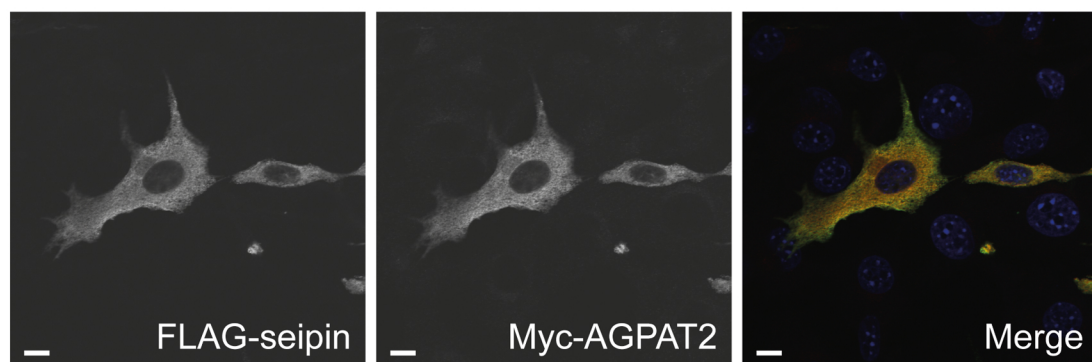

Supplement: Supplementary file 1 — Figure S1. (A) As a control for the BiFC analysis shown in Figure 2A 3T3-L1 preadipocytes were induced to differentiate and immediately co-transfected with FLAG-seipin-Yn and Yc-myc-AGPAT2. Cells were fixed after three days of differentiation, and anti-FLAG or anti-Myc antibodies in combination with Alexa Fluor 594 secondary antibodies were used to immunodetect seipin-Yn or AGPAT2-Yc respectively. No reconstituted YFP signal could be detected in any cells. In the lower panels cells were co-immunostained for FLAG-seipin-Yn and Yc-myc-AGPAT2-Yc. Scale bars, 10 μm. (B) control for cells transfected for Figure 2D,E. 3T3-L1 preadipocytes were induced to differentiate and immediately co-transfected with FLAG-seipin and myc-AGPAT2-Yc as in Figure 2D. Cells were fixed at day 3 of differentiation and stained for FLAG-seipin, myc-AGPAT2-Yc and DAPI to label nuclei. All cells expressing myc-AGPAT2-Yc co-expressed FLAG-seipin. Scale bars, 10 μm. [file mmc1.pdf]
